# Two new species of *Oreocharis* (Gesneriaceae) from Fan Si Pan, the highest mountain in Vietnam

**DOI:** 10.3897/phytokeys.94.21329

**Published:** 2018-01-29

**Authors:** Wen Hong Chen, Quang Hieu Nguyen, Run Zheng Chen, Tien Hiep Nguyen, Sinh Khang Nguyen, Van Tap Nguyen, Michael Möller, David J. Middleton, Yu-Min Shui

**Affiliations:** 1 Key Laboratory for Plant Diversity and Biogeography of East Asia, Kunming Institute of Botany, Chinese Academy of Sciences, 132 Lanhei Road, Kunming 650201, Yunnan, China; 2 Centre for Plant Conservation of Vietnam (CPC), Vietnam Union of Science and Technology Associations, 25/32 Lane 191, Lac Long Qua Road, Hanoi, Vietnam; 3 Karst Conservation Initiative of Yunnan, 132 Lanhei Road, Kunming 650201, Yunnan, China; 4 Institute of Ecology and Biological Resources, Vietnam Academy of Science and Technology, 18 Hoang Quoc Viet Road, Hanoi, Vietnam; 5 Royal Botanic Garden Edinburgh, 20A Inverleith Row, Edinburgh EH3 5LR, UK; 6 Herbarium, Singapore Botanic Gardens, National Parks Board, 1 Cluny Road, Singapore 259569

**Keywords:** Biogeographical affinis, Sino-Himalayan forest subkingdom, Southeastern Asia, *Oreocharis* with yellow or orange flowers

## Abstract

Two new species of *Oreocharis* Benth. from Fan Si Pan, the highest mountain in Vietnam (Sa Pa) are described and illustrated. *Oreocharis grandiflora* W.H.Chen, Q.H.Nguyen & Y.M.Shui, is similar to *O. flavida* Merr. from Hainan province, China, but differs mainly by its larger and infundibuliform corolla, stamens adnate to the base of the corolla tube and stamens coherent in two pairs. The second, *Oreocharis longituba* W.H.Chen, Q.H.Nguyen & Y.M.Shui, is similar to *O. hirsuta* Barnett, endemic to northern Thailand, but mainly differs in its pubescence, coherent stamens and glabrous filaments.

## Introduction

Fan Si Pan is a species-rich diversity hotspot in Indochina, the flora of which is still incompletely known. Fan Si Pan (in Vietnamese: Phan Xi Păng), the highest mountain in Vietnam (3143 m elevation), is situated in the northwest of the country and its orogeny is linked to the Himalayan Mountain chain (Nguyen and Harder 1996; Tapponnier et al. 1990, 2001). It also has the highest recorded levels of biodiversity in Indo-China and is part of one of the 25 world’s biodiversity hotspots (Takhtajan 1986; Myers et al. 2000). With more than 100 years of collecting and research in Fan Si Pan, a rich flora of 1659 species in 723 genera and 228 families has been recorded (Nguyen and Nguyen 1998). According to the floristic subdivision of Eastern Asia, Fan Si Pan is floristically related to the Sino-Himalayan forest subkingdom (= Sino-Himalaya Floristic Region in the past) (Nguyen and Harder 1996, Wu and Wu 1996). Even after a century of research, Fan Si Pan still yields new species. Over the last few decades several new species have been described, such as *Abies fansipanensis* Xiang et al. and *Manglietia crassifolia* Vu et al., adding to our understanding of its floristic affinities (Xiang et al. 1997; Vu and Xia 2010; Vu et al. 2011).

The genus *Oreocharis* Benth. now includes over 90 species after its recent re-circumscription (Möller et al. 2011). Since then, several new taxa have been described from China and the genus now includes over 106 species (Möller et al. 2016). The genus is distributed predominantly in China, with few species in Thailand, Myanmar, Bhutan, NE India, Japan and Vietnam (Möller et al. 2011; Möller and Clark 2013; Möller et al. 2014, 2016). Most *Oreocharis* species occur in relatively restricted and geographically isolated localities with very few widely distributed, such as *O. aurea* Dunn, occurring from South Yunnan in China (type locality) to North Vietnam (Pellegrin 1930; Wang et al. 1990, 1998; Li 1991; Ho 2000). No new species of *Oreocharis* were described from Vietnam from 1908 until recently when three new species were discovered (Do et al. 2017; Chen et al. 2017).

**Figure 1.**
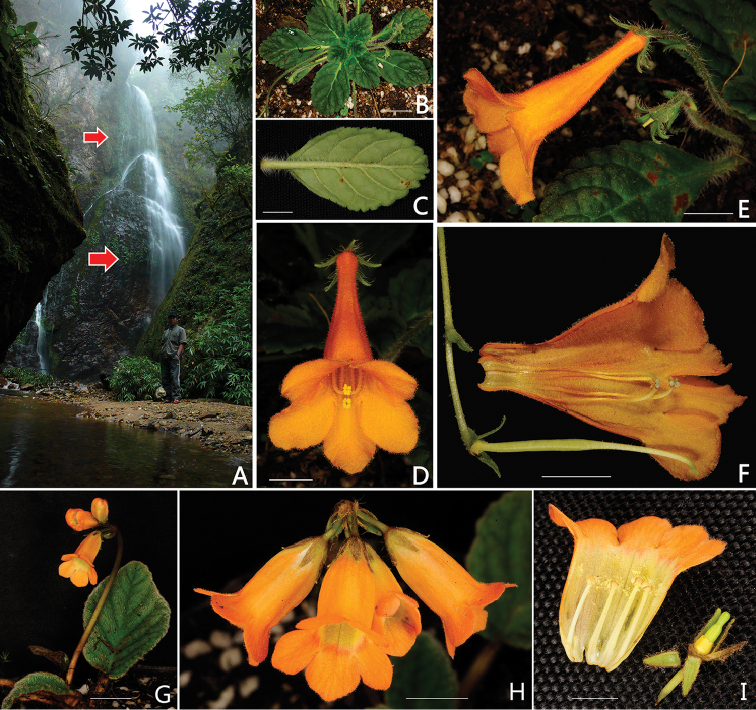
*Oreocharis grandiflora* W.H.Chen, Q.H.Nguyen & Y.M.Shui, sp. nov. (**A–F**) and *O. flavida* Merr. (**G–I**) **A** Habitat (red arrows indicate position of plants in the field) **B** Mature plant **C** Abaxial leaf surface **D** Front view of flower **E** Lateral view of flower **F** Opened corolla, pistil, disc and calyx **G** Plant **H** Inflorescence and open flowers **I** Dissected flower, showing corolla with free anthers, pistil, disc and calyx. Scale bars: **A, C–F** = 1 cm; **B,G** = 2 cm; **H, I** = 5 mm. All photographs by Yu-Min Shui.

**Figure 2.**
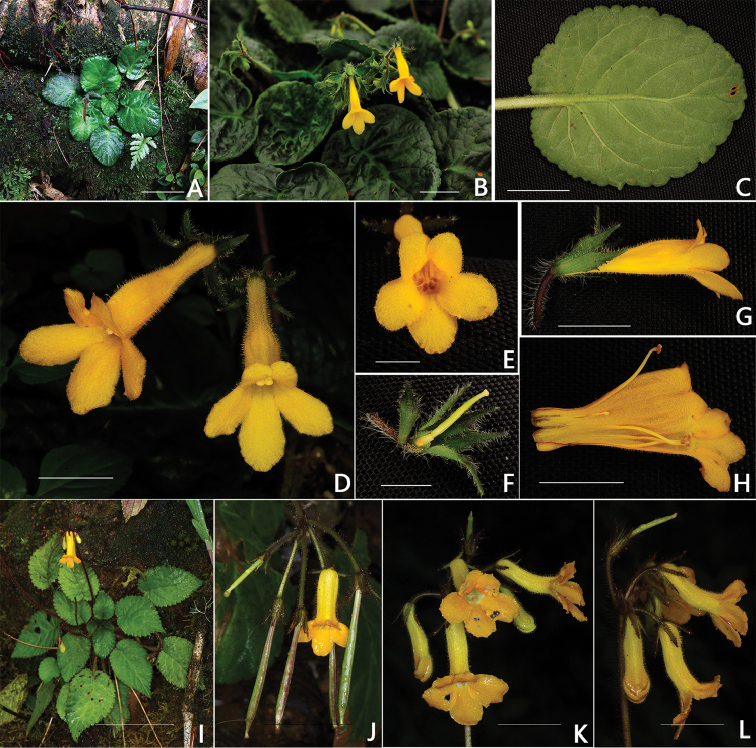
*Oreocharis longituba* W.H.Chen, Q.H.Nguyen & Y.M.Shui, sp. nov. (**A–H**) and its most similar species, *Oreocharis hirsuta* Barnett (**I–L**) **A** Habitat **B** Mature plant **C** Abaxial leaf surface **D, E** Front view of flower **F** Pistil with immature stigma, disc and calyx **G** Lateral view of flower **H** Opened corolla **I** Mature plant **J** Flower and fruits **K** Front view of flowers **L** Side view of flowers. Scale bars: **A, I** =5 cm, **D–H** = 1 cm; **J, K, L**= 2 cm. **A–H** photographs by Yu-Min Shui, **I–L** by Preecha Karaket of *Middleton et al. 4550*.

During a joint Sino-Vietnamese botanical survey in Fan Si Pan in November 2012, two of the authors (QHN and YMS) collected several specimens of Gesneriaceae. These included two collections of fruiting specimens. From the vegetative habit and fruit characters, they were identified as belonging to *Oreocharis*. In September 2013, cultivated plants of the two collections produced flowers unlike any of the described species in the genus (Figs 1 and 2). After consulting the relevant literature from China and Vietnam (Barnett 1961; Ho 2000; Wang et al. 1990, 1998; Li and Wang 2004; Chen et al. 2017; Do et al. 2017), it was confirmed that the two species were new to science. On examination of other recent and historic unidentified collections from Vietnam, a number of other specimens of one of the species were also found. Here, they are described and illustrated via photography and drawings.

## Taxonomy

### Oreocharis grandiflora

Taxon classificationPlantaePasseriformesParamythiidae

W.H.Chen, Q.H.Nguyen & Y.M.Shui sp. nov.

urn:lsid:ipni.org:names:77175492-1

Figs 1A–F , 3

#### Diagnosis.

This new species is similar to *O. flavida* in the orange colour of the corolla, but differs from the latter by its much larger corolla (3.3–3.6 cm long vs. 1.5–1.7 cm), the shape of the corolla tube (infundibuliform vs. campanulate) and the reniform anthers which are coherent in two pairs (vs. horseshoe-shaped and not coherent). The two species further differ by the narrowly oblong or elliptic leaf blades in the new taxon (vs. ovate-elliptic to broadly ovate), cuneate leaf base (vs. cordate to rounded), the glandular villous indumentum on the outer surface of the calyx lobes (vs. eglandular villous).

#### Type.

VIETNAM. Lao Cai, Sa Pa distr., Ta Phin cave, in secondary forests, on cliffs nearby waterfalls, 22°20'43.66"N, 103°46'30.48"E, 2017 m elevation, 30 October 2012, type specimen from a plant cultivated in an experimental greenhouse at Kunming Botanic Garden, 7 September 2013, *Y.M. Shui* et al. *B2013-550* (holotype, KUN!; isotype: Herbarium of the Centre for Plant Conservation, Vietnam Union of Science and Technology Associations, Hanoi!).

Perennial herbs. Leaves in basal rosette. Petiole 2.2–2.6 cm long, with dense white glandular hairs; leaf blade coriaceous, narrowly oblong or elliptic, 4–6 × 1.8–3.5 cm, adaxially and abaxially covered by white glandular hairs, more densely on veins, base narrowly cuneate, apex acute, margin crenate; lateral veins 4–5 on each side of the midrib, adaxially depressed, abaxially prominent. Inflorescences axillary, 1–4-flowered. Peduncles 6–12 cm long, with white glandular hairs; bracts 2, lanceolate, 5.6–6 × 1.1–1.2 mm, abaxially covered by white glandular hairs. Pedicel 1.5–1.8 cm long. Calyx 5-lobed from base, lobes equal, linear-lanceolate, 7–8 × 1.1–1.2 mm, entire, adaxially glabrous, abaxially with white glandular hairs. Corolla deep orange, slightly bilabiate, 3.3–3.6 cm long, inside pubescent, outside with white glandular short hairs; tube infundibuliform, 2–2.2 cm long, 2.7–3 mm in diam. at base and 8–9 mm in diam. at throat; adaxial lip 2-lobed, lobes suborbicular, 8.5–9 × 11–12 mm, apex obtuse; abaxial lip 3-lobed, lobes suborbicular, slightly equal, 13–14 × 8–9 mm, apex more or less rounded. Stamens 4, anthers coherent in two pairs, filaments adnate to base of corolla tube, adaxial stamens 2–2.2 cm long, abaxial stamens 2.6–2.8 cm long; filaments with white glandular hairs; anthers reniform, basifixed; staminode 1, adnate to base of corolla tube, 5–6 mm long. Pistil 3.1–3.5 cm long when mature; ovary cylindrical, 2–2.2 cm long, glabrous; style 1–1.3 cm long, with white glandular hairs; stigma 1, flattened with central depression. Disc ringlike, yellowish, 2–3 mm high. Capsule straight, cylindrical, 2.1–2.5 cm long.

#### Distribution, habitat and phenology.

This new species is endemic to Sa Pa, northern Vietnam and grows densely on cliffs by waterfalls along deep valleys in evergreen broad-leaved forests, at an elevation of around 1800–2010 m. Flowering from August to October and fruiting from September to October.

#### Etymology.

The species epithet refers to the large size of the flowers. Based on the authors’ observation and other relevant publications (Wang et al. 1990, 1998), the new species has one of the largest flowers in *Oreocharis*.

#### Conservation status.

This new species appears to be restricted to a very moist habitat in Sa Pa, Lao Cai Province, northern Vietnam. It grows on several steep cliffs at 1800–2100 m elevation by waterfalls with flowing water throughout the year (Fig. 1A). It flowers during the rainy season (September to October), during which the locality is inaccessible. This is likely the reason why it had not previously been discovered. It is naturally protected by its inaccessible habitat on the cliffs. According to our observations in the field, the two known populations harbour about 100 mature individuals in each. In fact, there are many waterfalls at this altitudinal range and, thus, the real number of populations and individuals may be higher. Nevertheless, its unusually humid habitat might be affected by climate change-induced droughts. Overall however, the species has been classified as “Data Deficient” [DD] following IUCN Red List Categories and Criteria (IUCN 2012).

#### Additional specimens examined.

VIETNAM. Lao Cai, Sa Pa distr., Ta Phin cave, in secondary forests, 22°20'43.66"N, 103°46'30.48"E, 2017 m elevation, 30 October 2012, in fruit, *Q.H.Nguyen, T.H.Nguyen, Y. M. Shui, Y. K. Sima, S. X. Yang, Z. Zhou, J. Liu CK687* (KUN!, Herbarium of the Centre for Plant Conservation, Vietnam Union of Science and Technology Associations, Hanoi!).

#### Notes.

This new species resembles *Oreocharis flavida*, but differs in the characters in Table 1 (see also Fig. 1). Additionally, the corolla size range is larger than any other species in the former delimitation of *Oreocharis*. In size and shape, the corolla of the new species resembles that of *Oreocharis ronganensis* (K.Y. Pan) Mich. Möller & A. Weber (formerly *Ancylostemon ronganensis* K.Y. Pan), but in the latter the corolla is pink, not deep orange. This is a rare colour in *Oreocharis* *s.l.*, since only about six of the >106 species have a corolla of such an intensely deep orange colour.

**Table 1.** Morphological comparison between *Oreocharis grandiflora* sp. nov. and *O. flavida* Merr.

Character	*O. grandiflora* sp. nov.	*O. flavida*
**Petiole**	2.2–2.6 cm long, white long glandular hairs	13–16 cm long, densely pale brown villous or woolly
**Leaf blade**	narrowly oblong or elliptic, 4–6 × 1.8–3.5 cm, adaxially and abaxially glandular, densely glandular on veins	ovate-elliptic to broadly ovate, 4–10 × 2–7.2 cm, adaxially densely pubescent, abaxially densely brown woolly, more densely along veins
**Leaf base**	cuneate	cordate to rounded
**Peduncle**	densely glandular	densely pale brown woolly
**Calyx**	outside glandular	outside eglandular villous
**Corolla**	3.3–3.6 cm long, outside white glandular	1.5–1.7 cm long, outside sparsely pubescent
**Corolla tube**	infundibuliform	campanulate
**Corolla lips**	adaxial lobes 8.5–9 × 11–12 mm, apex obtuse; abaxial lip 3-lobed, lobes 13–14 ×8–9 mm	all lobes slightly equal, 3–6 × 3–5 mm.
**Stamens**	4, anthers coherent in two pairs; anthers reniform	4, anthers not coherent; anthers horseshoe-shaped
**stigma**	1	2
**disc**	2–3 mm tall	1 mm tall

### Oreocharis longituba

Taxon classificationPlantaePasseriformesParamythiidae

W.H.Chen, Q.H.Nguyen & Y.M.Shui sp. nov.

urn:lsid:ipni.org:names:77175493-1

Figs 2A–H , 4

#### Diagnosis.

This new species is similar to *O. hirsuta* Barnett from Thailand, but differs from it in its pubescent petioles (vs. hirsute), (sub)orbicular leaves (vs. narrowly ovate or lanceolate), rounded leaf apex (vs. acute to short acuminate), crenate leaf margin (vs. bi-serrate), narrowly infundibuliform corolla tube (vs. tubular), anthers coherent in pairs (vs. free) and glabrous filaments (vs. hirsute).

#### Type.

VIETNAM. Lao Cai, Sa Pa distr., Ta Phin cave, in secondary forests, 22°20'54.48"N, 103°46'12.98"E, 1879 m elevation, 30 October 2012, type specimens from plants cultivated in an experimental greenhouse at Kunming Botanic Garden, 7 September, 2013, *Y.M. Shui* et al. *B2013-551* (holotype, KUN!; isotype, Herbarium of the Centre for Plant Conservation, Vietnam Union of Science and Technology Associations, Hanoi!).

Perennial herb. Leaves in basal rosette. Petiole 4–7 cm long, densely long pubescent; leaf blade (sub)orbicular, 3–9 × 2.4–8.9 cm, adaxially sparsely hirsute, abaxially pubescent, more densely so on venation, base cordate, apex rounded, margin crenate; lateral veins 5–6 pairs, adaxially depressed, abaxially prominent. Inflorescences axillary, 1–2-flowered. Peduncles 8–11 cm long, densely white villous; bracts 2, linear-lanceolate, 5–22 × 0.7–1.2 mm, adaxially subglabrous, abaxially pubescent; pedicel 1.8–2 cm, pubescent. Calyx 5-parted almost from base, segments linear-lanceolate, 8–15 × 1–4 mm, margin dentate, adaxially glabrous, abaxially white hispid. Corolla yellow, bilabiate, 3–3.5 cm long, inside pubescent, outside white glandular; tube narrowly infundibuliform, 2–2.5 cm long, 3–3.5 mm in diam. at base and 6–7 mm in diam. at throat; adaxial lip 6.5–7 mm long, 2-lobed, lobes suborbicular, 3.3–3.5 × 3.5–3.8 mm, apex obtuse; abaxial lip 3-lobed, lobes sub-oblong, almost equal, 8–10 × 6–8 mm, apex obtuse. Stamens 4, anthers coherent in two pairs, adaxial stamens 5–7 mm long, adnate to corolla tube 1.2–1.5 mm from base, abaxial stamens 7.5–8 mm long, adnate to corolla tube 1–1.2 mm from base; filaments glabrous; anthers round, basifixed, dehiscing longitudinally; staminode 1, 2.5–3 mm long, adnate to corolla tube 6–7 mm from base. Pistil 1.7–2.1 cm long when mature; ovary cylindrical, 1.2–1.4 cm long, glabrous, 2-locular; style 5–7 mm long, white pubescent; stigma 1, flattened with a central depression. Disc cylindrical, yellowish, 2.8–3 mm high, margin shallowly dentate. Capsule straight, cylindrical, 3–5 cm long. Seeds oblong, 1.1–1.2 mm long.

#### Distribution, habitat and phenology.

This new species is also endemic to Sa Pa, northern Vietnam and grows widely scattered on wet ground along road sides or along streams in evergreen broad-leaved forests, at an elevation of 1700–1890 m. Flowering from August to September and fruiting from September to October.

#### Etymology.

The species epithet refers to the unusually long length of the corolla tube in *Oreocharis*.

#### Conservation status.

Endangered EN B2ab (iii), following IUCN (2012) guidelines. This is based on an EOO of < 35 km^2^, being known from fewer than five populations and with disturbed locality.

#### Additional specimens examined.

VIETNAM. Lao Cai, Sa Pa distr., Kuoang Village, 22°28'43.66"N, 103°47'41.5"E, 1700 m elevation, growing on humus soil in wet and shady places, 11 September 2005, *X. P. Vu, D. H. Duong, V. D. Nguyen, Q. B. Nguyen, T.D.Nguyen, R. de Kok, G. Bramley, G. Challen, M. Vorontsova HNK 58* (K!); Sa Pa, Ta Phin cave, in secondary forests, 22°20'54.48"N, 103°46'12.98"E, 1879 m elevation, 30 October 2012, in fruit, *Q. H. Nguyen, T.H. Nguyen, Y. M. Shui, Y. K. Sima, S. X. Yang, Z. Zhou, J. Liu CK670* (KUN!, Herbarium of the Centre for Plant Conservation, Vietnam Union of Science and Technology Associations, Hanoi!); Sapa distr., the path to Fanxipan from Ton Station, 22°20'01"E, 103°46'47.8"E, 2000 m elevation, 10 August 2007, in fruit, *N. V. Du, P. Wharton & B. Wynn-Jones 10* (K!); Tonkin, route de Chapa à la garderie du Col de Lo Qui Ho, 1800 m elevation, September 1929, in flower, *P. A. Pételot 5177* (P: P03934211!; P04079324!; P03511246!); Col de Lo Qui Ho, elev. 2000 m, August 1933, in flower, *P. A. Pételot 7247* (P: P03934227); Col de Lo Qui Ho, elev. 1900, 16 August 1926, in flower, *Poilane 12965* (P: P04079331).

#### Notes.

A previous collection of this species, *X.P. Vu* et al. *HNK 58*, K!, had been identified as *Oreocharis hirsuta*, a species from Thailand that demarcates the southernmost distribution of the genus (Barnett 1961; Möller et al. 2011). When comparing the specimens studied here with type material and recent collections of *O. hirsuta* in the herbaria of the Royal Botanic Gardens, Kew (K) and the Royal Botanic Garden Edinburgh (E), it was found that they can be morphologically differentiated (Table 2; Figs 2I–L). Earlier collections by Pételot and Poilane in Vietnam and deposited in the Muséum National d’Histoire Naturelle in Paris (P), remained unnamed until now. These collections were made near Chapa at Lo Qui Ho, a station on the slopes near the summit of Fan Si Pan).

**Table 2.** Morphological comparison between *Oreocharis longituba* sp. nov. and *O. hirsuta* Barnett.

Character	*O. longituba* sp. nov.	*O. hirsuta*
**Petiole**	pubescent	hirsute
**Leaf blade**	(sub)orbicular, margin crenate	narrowly ovate or lanceolate, margin bi-serrate
**Peduncle**	densely white villous	hirsute
**Calyx**	0.8–1.5 cm long, abaxially hispid	3.4–7.5 mm long, abaxially hirsute
**Corolla**	3–3.5 cm long, inside pubescent	1.9–2.5 cm long, inside glabrous
**Corolla tube**	2–2.5 cm long, 3–3.5 mm in diam. at base and 6–7 mm in diam. at throat	1.5–1.9 cm long, 4–5 mm in diam. from base to top
**Corolla lip**	lobes unequal	lobes more or less equal
**Stamens**	4, anthers coherent in two pairs; filaments glabrous; anthers round	4, anthers not coherent, glabrous; filaments hirsute; anthers oval
**Ovary**	1.2–1.4 cm long	5–5.5 mm long
**Disc**	2.8–3 mm tall	1–2 mm tall

**Figure 3.**
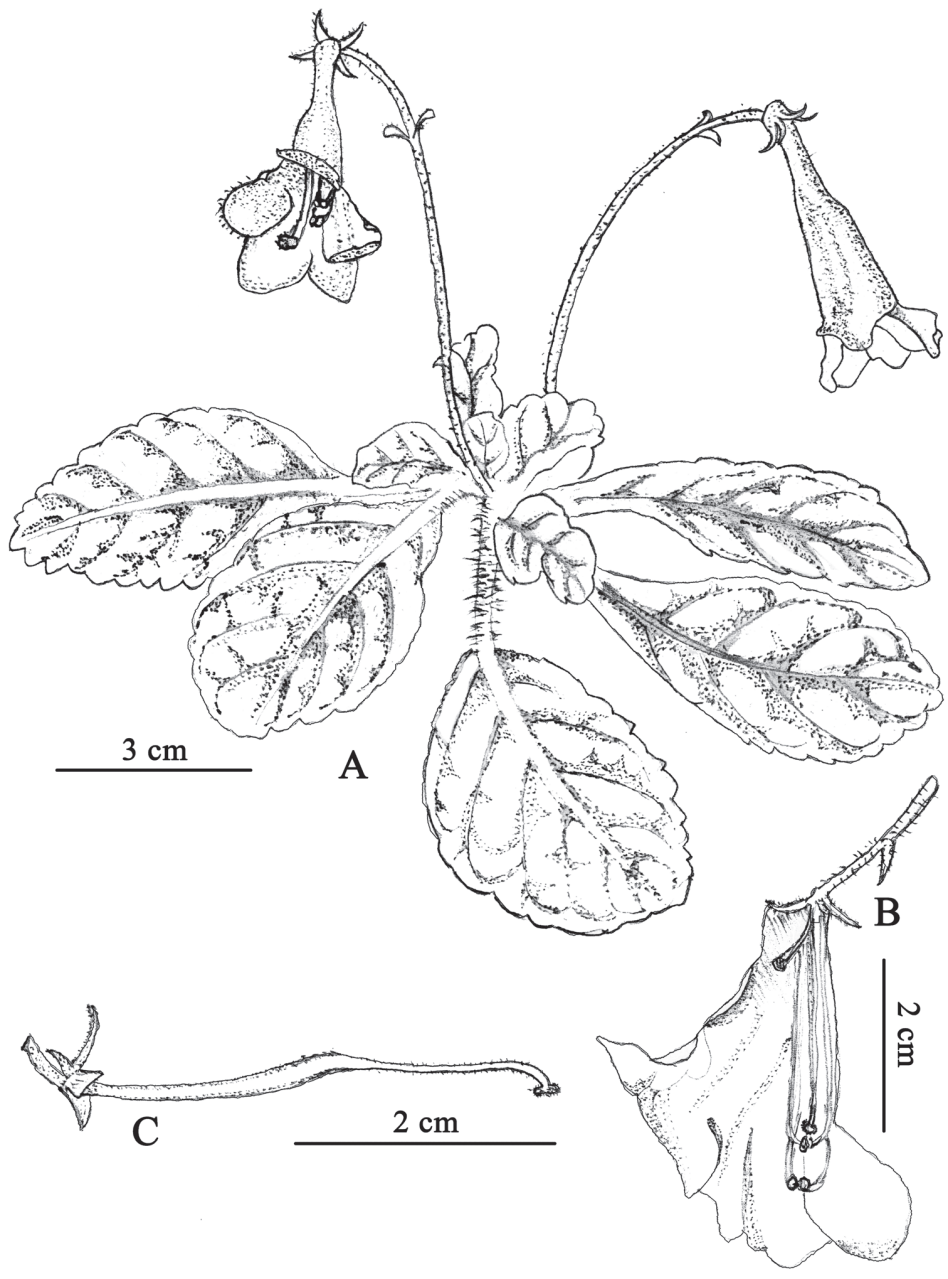
*Oreocharis grandiflora* W.H.Chen, Q.H.Nguyen & Y.M.Shui, sp. nov. (all drawings based on the holotype *Y.M. Shui* et al. *B2013-550* in KUN, drawn by Y.F. Shui) **A** Habit **B** Opened corolla showing corolla lobes and two pairs of stamens **C** pistil at stigma receptivity and calyx.

**Figure 4.**
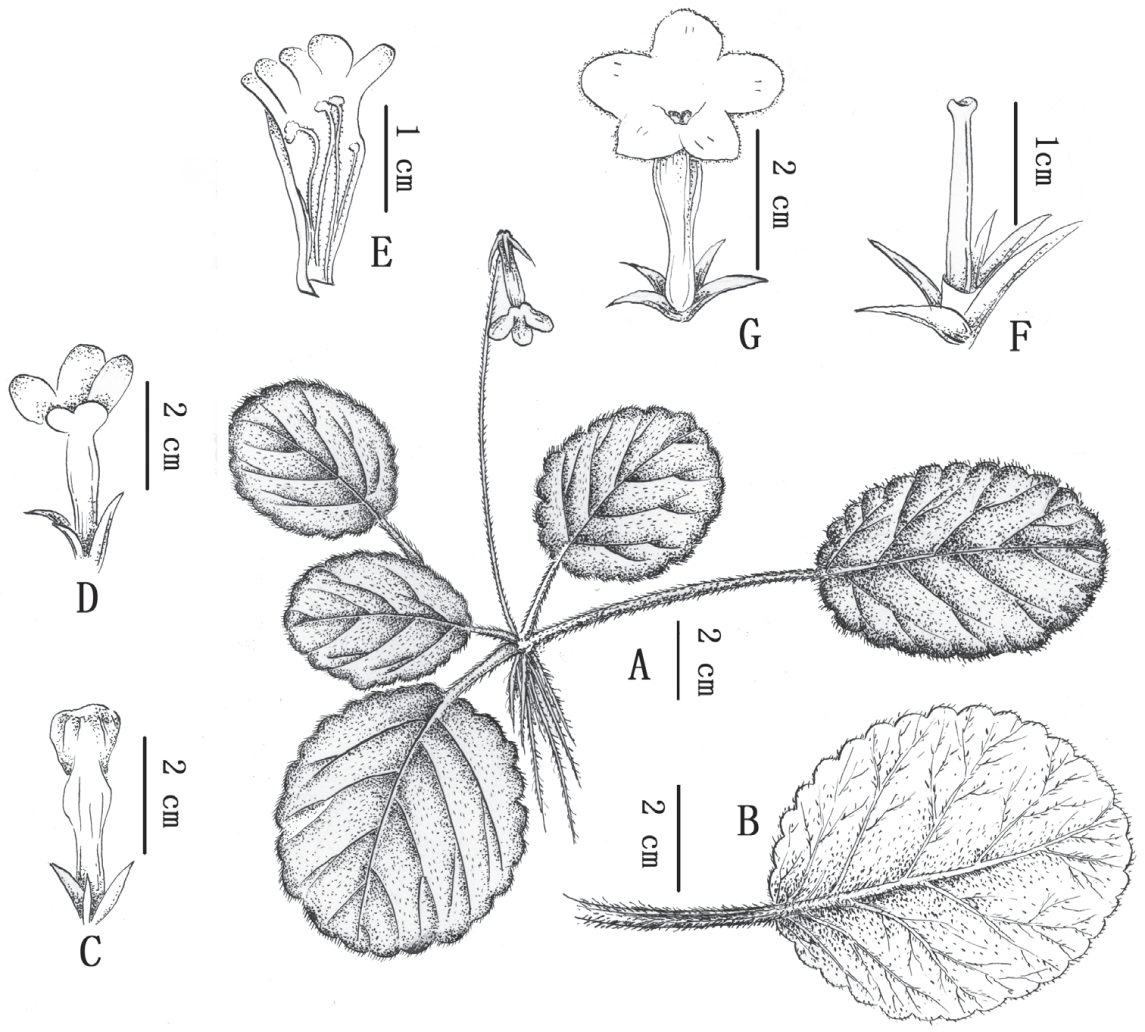
*Oreocharis longituba* W.H.Chen, Q.H.Nguyen & Y.M.Shui, sp. nov. (all drawings based on the holotype *Y.M. Shui* et al. *B2013-551* in KUN, drawn by J.X. Liu). **A** Habit **B** Abaxial leaf surface **C** Opening flower from below showing the inflated part near the distal end of the corolla tube **D** Open flower from above **E** Opened corolla showing two pairs of stamens **F** Pistil (immature at male stage), disc and calyx **G** Front view of flower.

With its long corolla tube up to 2.5 cm, *O. longituba* has the longest tube amongst the yellow flowered species with infundibuliform corollas in *Oreocharis*. It is also the only species with coherent anthers amongst species in the previous, narrower concept of *Oreocharis*. In the current wider delimitation of *Oreocharis*, the corolla tube is more similar in shape, though not in size, to those species previously placed in *Ancylostemon* Craib and *Paraisometrum* Wang (Wang et al. 1990, 1998; Weitzman et al. 1997; Chen et al. 2014).

## Supplementary Material

XML Treatment for Oreocharis grandiflora

XML Treatment for Oreocharis longituba
